# Global patterns and impacts of El Niño events on coral reefs: A meta-analysis

**DOI:** 10.1371/journal.pone.0190957

**Published:** 2018-02-05

**Authors:** Danielle C. Claar, Lisa Szostek, Jamie M. McDevitt-Irwin, Julian J. Schanze, Julia K. Baum

**Affiliations:** 1 Department of Biology, University of Victoria, Victoria, British Columbia, Canada; 2 Earth and Space Research, Seattle, Washington, United States of America; Leibniz Centre for Tropical Marine Research, GERMANY

## Abstract

Impacts of global climate change on coral reefs are being amplified by pulse heat stress events, including El Niño, the warm phase of the El Niño Southern Oscillation (ENSO). Despite reports of extensive coral bleaching and up to 97% coral mortality induced by El Niño events, a quantitative synthesis of the nature, intensity, and drivers of El Niño and La Niña impacts on corals is lacking. Herein, we first present a global meta-analysis of studies quantifying the effects of El Niño/La Niña-warming on corals, surveying studies from both the primary literature and International Coral Reef Symposium (ICRS) Proceedings. Overall, the strongest signal for El Niño/La Niña-associated coral bleaching was long-term mean temperature; bleaching decreased with decreasing long-term mean temperature (n = 20 studies). Additionally, coral cover losses during El Niño/La Niña were shaped by localized maximum heat stress and long-term mean temperature (n = 28 studies). Second, we present a method for quantifying coral heat stress which, for any coral reef location in the world, allows extraction of remotely-sensed degree heating weeks (DHW) for any date (since 1982), quantification of the maximum DHW, and the time lag since the maximum DHW. Using this method, we show that the 2015/16 El Niño event instigated unprecedented global coral heat stress across the world's oceans. With El Niño events expected to increase in frequency and severity this century, it is imperative that we gain a clear understanding of how these thermal stress anomalies impact different coral species and coral reef regions. We therefore finish with recommendations for future coral bleaching studies that will foster improved syntheses, as well as predictive and adaptive capacity to extreme warming events.

## Introduction

Climate change poses an imminent threat to the persistence of the world's coral reefs. Anthropogenic ocean warming is fundamentally altering marine ecosystems [1], exacerbating chronic local stressors such as overfishing, eutrophication, and coastal pollution, and threatening the resilience of marine ecosystems [2,3]. With increasing anthropogenic stressors, many coral reef ecosystems have transitioned from “safe operating spaces”–ecosystem states that are resilient to periodic stress events—towards “zone(s) of uncertainty” in which natural variability limits our prediction of ecosystem response, and “zone(s) of high risk” in which the ecosystem and its associated functions are already degraded [4]. Global surface warming manifests not only as gradual increases in overall temperature, which are predicted to exceed 2°C by 2100 [5], but also as intense pulse heat stress events such as the warm phase of the El Niño Southern Oscillation (ENSO). ENSO is a quasi-periodic fluctuation in oceanographic and atmospheric conditions, which transitions among El Niño, neutral, and La Niña conditions. El Niño is associated with increases in sea surface temperature (SST) that are primarily centered in the Central and Eastern Tropical Pacific Ocean [6]. Short-term positive warm temperature events caused by both El Niño and La Niña have instigated coral bleaching, so including both types of events allows us to investigate ENSO-related warming both in the Eastern/Central Pacific and in the Western Pacific, respectively. Major El Niño events have triggered three global coral bleaching events over the past four decades, the most intense of which unfolded over the course of 2015 and 2016 [7]. Extreme El Niño events are predicted to double in the future due to greenhouse warming [8], and the frequency of severe coral bleaching events is expected to increase even under moderate warming scenarios [9]. This projected increase in pulse warming events further threatens reefs which are already facing a multitude of stressors.

Short-term thermal stress events, such as El Niño, primarily impact corals by inducing coral bleaching. Under normal conditions, the coral animal lives in symbiosis with endosymbiotic *Symbiodinium* (previously, zooxanthellae), single-celled algae that reside in the coral's tissue and provide metabolic products necessary for coral survival [10,11]. Coral bleaching results from a loss of *Symbiodinium*, which can occur under stressful conditions as the symbiosis breaks down leading to the coral losing pigmentation as *Symbiodinium* lose photosynthetic functionality and are ejected from the coral tissue [12]. If the symbiosis is not reestablished before the coral is depleted of metabolic products and energy reserves, subsequent mortality can occur [13]. Coral bleaching can occur in response to a variety of environmental stressors, the most documented of which is increased water temperatures [14]. Substantial natural gradients of environmental stressors, including sea surface temperature regimes, exist at varying spatial and temporal scales [15, 16], and corals begin to bleach when ocean temperatures exceed local thermal thresholds. Coral bleaching severity and the extent of subsequent coral mortality is highly variable both within and across regions. Moreover, coral bleaching can affect any reef, occurring not only in areas with coastal human populations but also on remote reefs [17] and in protected areas [18]. While bleaching variability may be partially attributed to coral species differences, many other factors appear to influence the magnitude of changes in the coral reef community after pulse warming events.

Coral bleaching and mortality caused by extreme El Niño/La Niña events can induce catastrophic changes in foundational coral reef ecosystem structure [19, 20]. The impact of extreme El Niño events on coral communities worldwide has been observed and quantified since the early 1980s [21–23]. Notable regional examples include estimates of up to 95% coral mortality in some locations in the Eastern Pacific [24] and nearly 100% local coral mortality at some sites in Indonesia [25] during the 1982–83 El Niño event, and up to 90% coral mortality on individual shallow Indian Ocean reefs during the 1997–98 El Niño event [26]. Many early studies and bleaching reports were based upon underwater visual observations of bleaching severity and coral mortality. When conducted without replication, visual estimates can preclude quantitative meta-analysis because they may be prone to intra-observer variability and may not include statistical measurements such as error (e.g. standard deviation, standard error, or confidence intervals) necessary for many quantitative analyses.

Depending on local conditions and the frequency of thermal stress, there is evidence that coral reefs can recover, even after an extreme El Niño/La Niña event [23,27–29]. Despite the potential for recovery, the effects of a single El Niño/La Niña event can be permanent. For example, one reef-building hydrocoral became locally extinct, while another was driven to probable extinction as a direct result of the 1982/1983 El Niño event [30], and Panamanian reef structure damaged by the 1982/1983 El Niño had not returned to pre-El Niño levels nearly twenty years later [31]. Even when the damage is not permanent, reefs can take more than a decade to recover from the impacts of an intense El Niño [32]. Despite these widely recognized and extensively cited effects of ENSO-related pulse warming events, a quantitative global analysis of the effects of El Niño/La Niña on coral communities has not yet been conducted. Our research builds upon previous reviews which investigated the impact of single El Niño events on coral reefs [33–35], differential bleaching of corals between two El Niño events in one region [36], and the long-term recovery of coral reefs after El Niño [37].

Here, we address three questions of relevance to understanding climate change impacts on coral reefs: 1) How much coral bleaching and mortality have been observed and documented during previous El Niño/La Niña events?, 2) Does the coral stress metric Degree Heating Weeks (DHW) accurately predict El Niño/La Niña-driven changes in coral bleaching and cover on a global scale, and what other factors are influential?, and 3) How does the 2015–2016 El Niño event compare to prior events in terms of severity (maximum DHW) and geographic extent? We conducted a global meta-analysis to quantify the effects of El Niño/La Niña events on coral communities. Although it is widely accepted that the increase of SST that results from El Niño/La Niña events induces coral bleaching, the consistency of the effects of El Niño/La Niña fluctuations on coral bleaching and mortality has yet to be quantified through multiple ENSO oscillations at a global scale. To evaluate the relationships between DHW and coral bleaching and mortality, we build upon previous research (Box 1) using the Reynolds OI Level 4 AVHRR 0.25° sea surface temperature product to compute a comprehensive coral heat stress data set. This method allows for calculation of remotely-sensed coral heat stress for any date since 1982 using a consistent data set and climatology, allowing comparison of coral heat stress indices and response throughout the majority of published El Niño/La Niña-related coral bleaching events.

**Box 1. Degree Heating Week (DHW) products and coral bleaching prediction**

Understanding how El Niño- and La Niña-related heat stress affects coral reefs at a global scale requires quantification of the magnitude of thermal stress for each reef location. Quantification of thermal stress on coral reefs using satellite observations began with the definition of ocean "hot spots" [38] and in situ validation of satellite temperature observations at the reef scale [39]. In tandem with the development of "hot spot" analyses, researchers developed the concept of Degree Heating Weeks (DHW) as a cumulative metric of thermal stress on coral reefs [19,40]. The US National Oceanographic and Atmospheric Administration (NOAA) Coral Reef Watch Program (CRW) further developed these methods, using high-resolution (9km satellite pixel) HotSpot anomaly mapping [41] which improved correlation of satellite temperature observations with in situ measurements of coral reef temperature fluctuations [42]. NOAA CRW DHW products now include both an updated 50-km product [43], and a newly-released 5-km product [44]. The NOAA CRW products have been successful in detecting many coral bleaching events around the globe (e.g. [45]; [46]), and can now be used to forecast upcoming thermal stress on coral reefs [47]. Despite these major developments in understanding and predicting thermal stress and coral reef bleaching, a study that compared two DHW metrics to ReefBase bleaching reports found poor congruence between DHW magnitude and bleaching events both expected and observed, which was attributed to localized temperature variability and related coral adaptation [48]. The extent of coral bleaching in a given location has been suggested to vary in relation to historical variability in maximum SST and local climatological maximums, although lack of adequate bleaching reports has hindered our understanding of these relationships and of global coral bleaching trends [49]. Most recently, nowcasting and predictive tools have allowed managers to prepare for and respond to coral bleaching events, and cutting-edge climatological heat stress models and analyses are continually being developed at the US NOAA CRW program [50] and the Australian Bureau of Meteorology [51]. As local and regional coral bleaching reports are increasingly quantified, analyzed, and published, we have the ability to better understand how events such as El Niño and La Niña affect coral reefs at a global scale.

## Methods

### Literature search and data extraction

In order to identify primary literature regarding the effects of ENSO events (El Niño and La Niña) on coral health, we conducted a systematic literature search using all databases in ISI Web of Science using the following search terms: (coral*) AND (mortal* OR bleach* OR cover* OR health*) AND (El Niño OR El Nino OR ENSO). We performed the search first on December 3, 2014, and updated it on March 20, 2015 and again on August 27, 2016. We performed a second related search in ISI Web of Science using the search terms: (coral*) AND (mortal* OR bleach* OR cover* OR health*) AND (La Niña OR La Nina) on September 16, 2016. Using a database search method (such as the ISI Web of Science keyword search employed in this study) is a common meta-analysis approach ([52]). In order to access relevant papers that were not published in the primary literature, we also queried the International Coral Reef Symposium (ICRS) Proceedings on ReefBase in three separate searches, first using the term 'El Niño', second using the term 'bleaching', and finally using the term 'La Niña'. Additional primary literature was included from bibliographies of relevant reviews or from within meta-analyses. To avoid data duplication and pseudo-replication, we did not include data from any summary reports. Although this may have limited our usable data to some extent, many reports either contained data that appeared elsewhere in the primary literature or that otherwise did not pass our selection criteria. An overview of all reviewed and included publications is given in S1 Table, and all extracted data are available in S2 and S3 Tables and at https://github.com/baumlab/Claar_ElNinoMetaAnalysis (DOI: 10.5281/zenodo.1134085).

**Fig 1:**
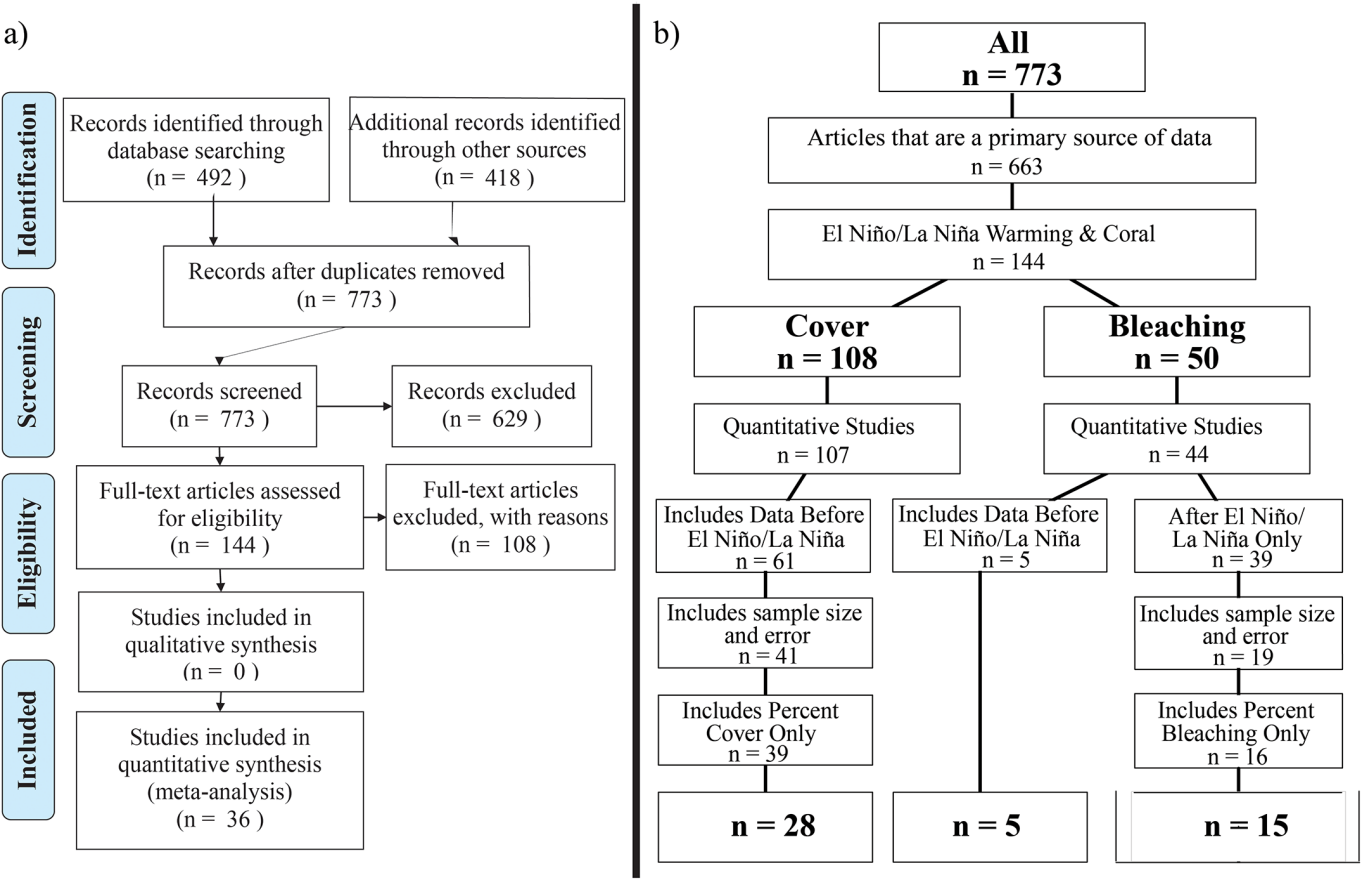
a) PRISMA (Preferred Reporting Items for Systematic Reviews and Meta-Analyses) 2009 flow diagram; and b) study-specific flowchart, both showing exclusion steps starting from studies returned from the full Web of Science and ICRS Proceedings literature search. Reviews and other meta-analyses, as well as secondary literature, or data which were repeated in more than one manuscript were excluded. Manuscripts which were not related to El Niño/La Niña warming, or otherwise not relevant to the current study were excluded. Relevant reviews were divided into manuscripts which address El Niño/La Niña-related changes in coral cover, and coral bleaching (n = 7 studies included both). Qualitative studies were removed, as they could not be included in analyses. Coral cover studies were then excluded if they did not include before-El Niño/La Niña data. Finally, studies were excluded if they did not include either sample size or a measurement of error, did not quantify a standardized metric, or were conducted more than 2 years after the El Niño/La Niña warming event. A PRISMA (Preferred Reporting Items for Systematic Reviews and Meta-Analyses) checklist is available in S1 Fig.

We then systematically evaluated each paper returned from our searches (Fig 1). We first read the abstract to determine the relevance of the paper to our research questions. If deemed relevant (i.e. the focus of the study was coral responses to an El Niño/La Niña event), we then examined the full text to evaluate if the data presented could be extracted and utilized. Selected studies had to: 1) provide information on coral responses (i.e. changes in bleaching or coral cover) to El Niño or La Niña including the sample size of the study and some measure of the variance (i.e. standard deviation or error, 95% confidence intervals), and 2) compare the coral responses before and during an El Niño/La Niña event or before and after an El Niño/La Niña event. Note that for coral bleaching we also accepted studies that had not reported the extent of bleaching prior to the heat stress event. Due to our exclusion criteria, we included only field-based studies, and we did not include any laboratory manipulation studies. We obtained the dates of El Niño/La Niña events from the Australian Government Bureau of Meteorology (http://www.bom.gov.au/climate/enso/outlook/) and El Niño/La Niña data was accepted from the beginning of El Niño/La Niña heat stress up to two years after the end of the El Niño/La Niña event. It is beyond the scope of this manuscript to evaluate long-term recovery of coral bleaching and cover (see [31] for an excellent review), so articles were also rejected if they quantified coral bleaching and recovery two years or more after the conclusion of the El Niño/La Niña event. Other reasons for rejecting an article included inaccessibility (e.g. not available through Web of Science, Google Scholar, ResearchGate, or author websites) or containing exclusively secondary data (e.g. reviews, meta-analyses).

For each of the studies that met our selection criteria, we extracted the data required to calculate effect size (i.e. mean, variance, and sample size). For studies with time-series data, we defined the before value as the data point closest to the commencement of the El Niño/La Niña event, and the El Niño/La Niña value as data collected from the peak of the El Niño/La Niña event until two years after the El Niño/La Niña event. In cases where data was presented as a timeline, we extracted the earliest data point corresponding to El Niño/La Niña impact. If data were presented exclusively in a graph, we used the software GraphClick [53] to obtain values from the figures. For studies that reported sampling error graphically, but the error bars were too small to be measured, we substituted in a small value (0.1) so that these measurements could be included in downstream analyses. If studies included both during (i.e. start of warming effects until six months after the peak) and post (i.e. six months to two years after peak warming) data points, we took each time frame as a separate data point. Where standard deviation was not reported, we calculated it from standard error or 95% confidence intervals. Since baseline coral bleaching outside of El Niño/La Niña events may not be zero, and some coral bleaching studies did not provide a before El Niño/La Niña estimate of bleaching (i.e. an estimate of local baseline bleaching), we simulated baseline bleaching levels using the conservative mean of 5% baseline bleaching (±15%SD). Very few studies included before-El Niño/La Niña bleaching incidence or severity (n = 6), so we expanded our search to analyze published studies which did not provide before-impact data, but only provided values that were during or after the event (n(after only) = 23) (Fig 1). We conducted two separate analyses on the full bleaching data set, with one using the extracted before-El Niño/La Niña values (with simulated before El Niño/La Niña values only for those instances where before-event bleaching was not reported), and the other using the simulated before-El Niño/La Niña values across the entire data set. We also extracted metadata from each selected paper including El Niño/La Niña year, coral taxonomic classification, sampling date, sampling depth, and latitude/longitude. In cases where geographical coordinates were not provided or were provided as the center of a region, we approximated the latitude and longitude from the text using Google Earth.

### DHW calculations based on a consistent 33-year climatology

To evaluate the effects of sea surface temperature (SST) on coral reefs, we extracted a coral stress temperature data set spanning the time period of this analysis. While there are a number of pre-compiled products that provide degree-heating-week (DHW) and instantaneous bleaching thermal stress [54], they are not readily available for the full period of 1982–2016. To facilitate this analysis, we derived a new data product from the Reynolds OI 0.25° Level-4 SST analysis. This product is based on blended data from the Advanced Very High Resolution Radiometer (AVHRR) missions as well as *in situ* data and is published by the Group for High Resolution Sea Surface Temperature (GHRSST) [55,56]. We chose this optimally interpolated product because it provides gapless, quality-controlled and in situ verified (and enhanced) estimates, which minimizes known issues with satellite-only datasets, such as erroneous cold pixels, gaps due to clouds, and land contamination. We believe that the GHRSST Level-4 AVHRR product provides the best compromise between robustness and resolution for the present analysis. We performed all computations in MATLAB using daily maps at a spatial resolution of 0.25° (approximately 25–30 kilometers).

The first parameter we derived from SST was the instantaneous bleaching thermal stress, hereafter 'hotspots'. Following the methods of [54], we computed hotspots by subtracting the maximum mean month (MMM) from the current SST at each grid point. We computed the MMM by taking the mean temperature of each month during the period 1982–2014 (the first and last complete years which were available at the time of this analysis) and finding the month with the highest mean temperature, that is, the warmest month of the monthly climatology. We ran this computation individually for each grid point in latitude and longitude, with the MMM generally occurring in the boreal summer months in the Northern Hemisphere and the austral summer months in the Southern Hemisphere. From the calculated hotspots, DHW were then computed using the following criteria (included if the hotspot reached or exceeded MMM+1°C), as defined by [54], but modified to accommodate the use of daily data. For example, if the daily values of *SST—MMM* are below zero for 11 weeks and then 0.2°C, 0.3°C, 0.5°C, 0.7°C, 0.9°C, 1.2°C, 1.3°C in the last week, only the values 1.2°C and 1.3°C would contribute to the DHW, for a total value of (1.2 + 1.3)/7, resulting in a DHW value of 0.36°C. The division by seven occurs due to the computation of DHW instead of degree-heating-days (DHD). To illustrate Time Lag, consider a point which has experienced significant heating 4 months prior to the sampling date. Here, latent effects may still be present, even though the current DHW may be zero. Therefore, in addition to current DHW, we return the previous maximum DHW and the time lag (in days) to it for inclusion in downstream analyses. To analyze a specific location and time, we return a number of additional output variables at each queried time and latitude/longitude (variables defined in Table 1; data accessible at https://www.CoralStress.org).

**Table 1:** Definition of derived variables included in our new data product. All variables are computed for a user-provided latitude/longitude and date, which can include any time point since 1984. With the exception of the first three months of 1982, we also calculate these same parameters for 1982–1983, although the beginning of usable data is in January 1982, so calculation of maximum DHW and time lag are restricted to within this window. Data are accessible at www.CoralStress.org.

Data Type	Abbreviation	Description
Current Degree Heating Week (DHW)	DHWnow	Current DHW calculated at the queried date, where any daily temperature exceeding the MMM by more than 1°C contributes to the DHW total, which is totaled over the previous 84 days (12 weeks).
Maximum DHW during El Niño	MaxDHW	Maximum DHW during the current year and the past three years.
Time Lag since Maximum DHW	TimeLag	Paired with MaxDHW. Time (in days) between queried date and maximum DHW. Only the time since the MaxDHW the corals have experienced are considered.
Current Sea Surface Temperature	SSTnow	Current sea surface temperature (SST) at the queried time.
Long-term Mean Temperature	SSTmean	Mean SST calculated over the period 1982–2014.
Long-term Temperature Variance	SSTvar	Variance of SST calculated over the period 1982–2014.
Long-term Temperature Standard Deviation	SSTstd	Standard deviation of SST calculated over the period 1982–2014.
Maximum Monthly Mean	MMM	Maximum monthly mean SST, computed by calculating the mean temperature of each month during the period 1982–2014 and selecting the month with the highest mean SST.
Month in which MMM occurs	MMMmon	Month in which the MMM occurs, ranges from January = 1 to December = 12.

### Calculation of effect sizes

We used the Metafor package in R [57,58] to calculate the effect size Hedges’ d [59] for both percent bleaching and percent cover. We utilized Hedges’ d because this commonly used effect size metric [60–62] allows the inclusion of reported zero-values, which occurred frequently in our data set (e.g. in cases where there was zero bleaching prior to the El Niño).

Equation 1: $d=((Mean_{1}-Mean_{2})/((\sqrt{(}(n_{1}-1)(s_{1}{)}^{2})+\frac{\left(n_{2}-1)(s_{2}{)}^{2}\right)}{n_{1}+n_{2}-2})*J)$ where Mean_1_ is the mean percent cover or bleaching cover during or after the El Niño/La Niña event, Mean_2_ is the mean percent cover or bleaching before the El Niño/La Niña event, s_1_ and s_2_ are the standard deviations, n_1_ and n_2_ are the sample sizes, and J is a correction for small sample size (Equation 2): *J* = 1 − (3/(4(*n*_1_ + *n*_2_)−1)). Variance for Hedges’ d is described by (Equation 3): *V*_*d*_ = ((*n*_1_ + *n*_2_)/(*n*_1_ * *n*_2_)) + (*d*^2^ / 2(*n*_1_ + *n*_2_)))

### Statistical analyses

We conducted all statistical analyses in R using the metafor package [57]. We first tested whether ENSO-associated warming increases coral bleaching or decreases coral cover by running each model without any moderator terms (i.e. random effects) to obtain the overall effect size. Here we used a random effects model, with individual estimates (e.g. by site or time point) nested within study number (i.e. publication) as the nested random effect, in order to account for heterogeneity amongst data points from within the same study and between studies, which can arise because of the consistent methodology, location, or species measured in an individual study. We used REML (restricted maximum-likelihood estimator) and we tested for within-group heterogeneity in the random effects model with the error heterogeneity estimate statistic (*Q*_*E*_) [63].

Next, to assess the factors influencing coral bleaching and coral mortality (measured as loss of coral cover), we used linear mixed-effects models with 'study' as the random effect and maximum DHW (MaxDHW), DHW time lag (i.e. time between maximum DHW and study measurement date; TimeLag), long-term temperature mean (SSTmean), and long-term temperature variance (calculated over the full 30-year AVHRR data set, SSTvar) as the moderators. Note that for coral bleaching we constructed two full models, one in which we simulated 'before' bleaching values for those studies missing this information and one in which we simulated 'before' bleaching values for the entire data set, as a sensitivity test of the effect of the 'before' simulation on our results. For each full model type, we used Akaike information criterion corrected for small sample size (AICc) to determine the best model by testing all possible combinations of moderators and all two-way interactions using the R package glmulti [64]. We also conducted reverse step-wise ANOVAs beginning with all possible combinations of moderators and continued simplifying the model until the minimum AICc was reached. For the step-wise ANOVAs we used MLE (maximum likelihood estimator) because it can be used to compare evidence weights (AICc) among models. In some cases, the top model had a similar AICc value as the next best models, we show these models in S3B and S3C Fig. We also conducted a comparison of up to one year post-event against model results for the full two years post-event. For each moderator retained, we extracted the effect size and 95% confidence intervals from the final top model to determine significance at the different levels. To test for model fit, we used a pseudo-R^2^ value calculated as follows: $\frac{\sum \sigma_{S}^{2}-\sum \sigma_{F}^{2}}{\sum \sigma_{S}^{2}}$ (Equation 4, generalized from [65]), where $\sigma_{S}^{2}$ is the variance component from the simple model (no moderators included), and $\sigma_{F}^{2}$ is the variance component from the full model (including all significant moderators). This pseudo-R^2^ value shows how much more variance is accounted for in the full model, compared to the simple model without moderators, or how much better the fit is when moderators are taken into account. Although this is not a perfect metric for goodness of fit, it is the best method currently available to assess goodness of fit for our models.

To evaluate our data for publication bias, we evaluated our models in funnel plots, and determined the fail-safe number for each overall model [66]. The fail-safe number represents the number of studies which would have to be added to the current meta-analysis in order to alter the significance of the overall effect size, with larger numbers providing support for the current result. Our fail-safe numbers for coral bleaching indicated that 118994 papers for the fully simulated before values and 48160 papers for partially simulated before values would need to be added in order to alter the statistical significance of the overall effect size. Similarly for coral cover, 46191 studies for coral cover would have to be added to alter the statistical significance of the overall effect size. These extremely large fail-safe numbers emphasize the strength of the relationship between El Niño/La Niña warming and coral decline.

## Results and discussion

### El Niño heat stress

**Fig 2:**
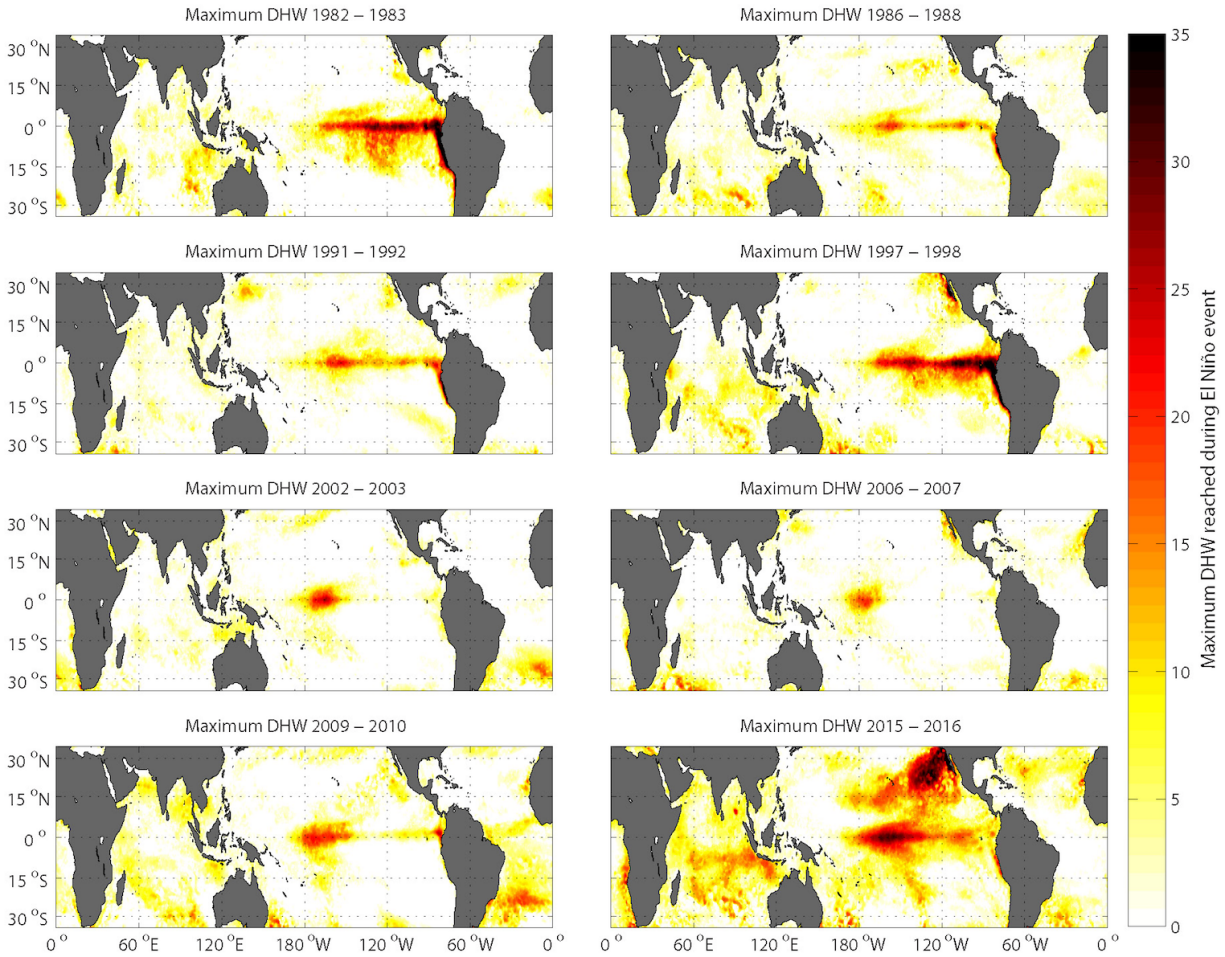
Maximum heat stress (DHW) for each reef location in the world (calculated at a 0.25° spatial resolution from AVHRR satellite data) during each of the eight El Niños that occurred in the past 35 years.

Comparisons of individual El Niño/La Niña thermal stress anomalies, using the consistent 33-year climatology in our new data product, reveal considerable differences in the overall intensity of heat stress during past El Niño events and in the locations of the maximum heat stress amongst these events (Fig 2 and S2 Fig). The El Niño events of 1982/1983 and 1997/1998, which were the largest prior to the 2015/2016 event [6,67–70], show a typical El Niño pattern, with maximum heating occurring along the equator and the western coast of South America (Fig 2 and S2 Fig). In contrast, the Central Pacific El Niño or “Modoki”, with maximum heating along the equator near the dateline, can be seen clearly in the 2002/2003 and 2009/2010 El Niño events (Fig 2 and S2 Fig). Additionally, global maps of the maximum cumulative El Niño for each reef location illustrate stark differences in severity and areal impact of El Niño-associated heat stress (Fig 2).

### Studies of El Niño/La Niña impacts on coral reefs

**Fig 3:**
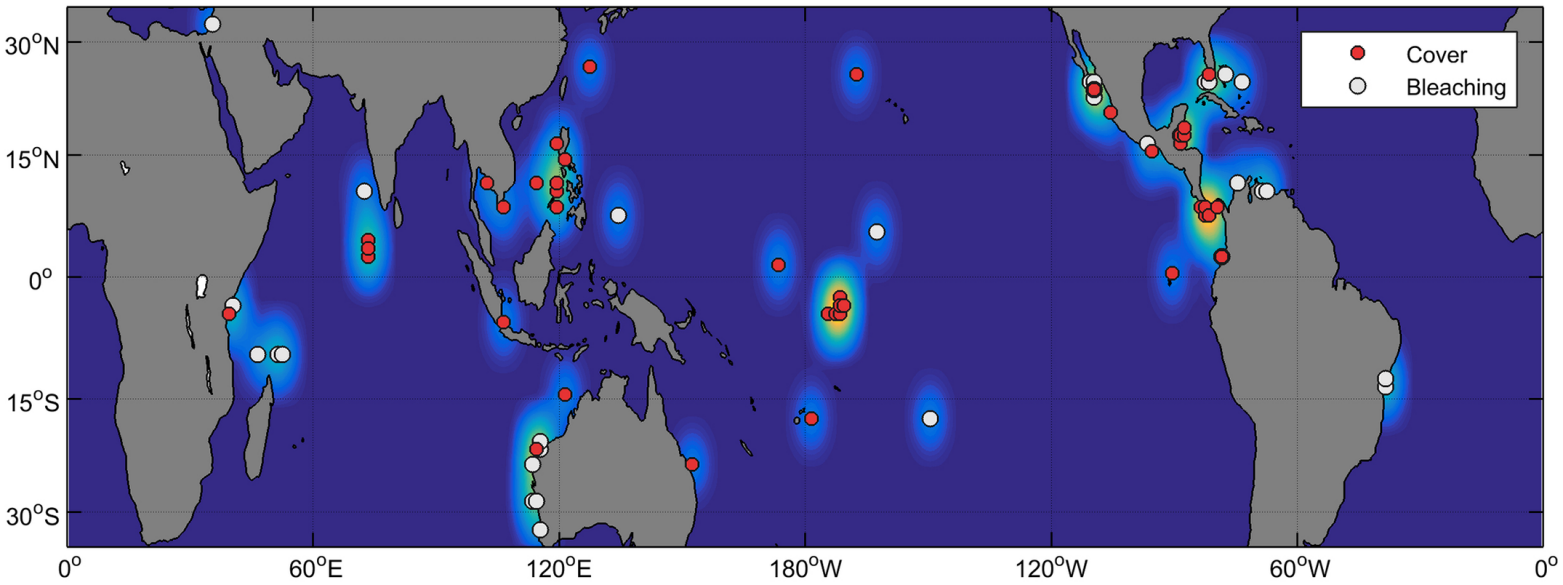
Study locations included in this global meta-analysis. Studies reporting changes in coral bleaching due to El Niño/La Niña warming are marked in white, and studies reporting changes in coral cover due to El Niño/La Niña warming are marked in red. The background color scale represents the number of data points that were extracted from each location. Data from non-El Niño/La Niña bleaching events, and from papers excluded from this meta-analysis are not included on this map.

Our literature search identified a total of 773 unique publications, of which 36 fit all of our search criteria. From the original articles returned from the searches, only 144 articles addressed coral bleaching or cover losses in relation to ENSO-associated warming (Fig 1). Many of the initially-excluded studies were returned from our search but were irrelevant to the current meta-analysis, such as analyses of coral skeletal composition for reconstruction of historic climate. There is some overlap between coral bleaching papers (n = 50), and coral cover papers (n = 108) when split from relevant papers (n = 144), because a few studies (n = 7) provided data on both coral bleaching and coral cover. After all exclusion steps were complete, there were 5 coral bleaching studies which included "before bleaching" data, 15 coral bleaching studies which included data only during or after the bleaching event, and 28 coral cover studies which included all necessary attributes for analysis (Fig 1). In total, 6 studies reported on coral bleaching associated with ENSO, and 30 studies reported on coral cover losses associated with El Niño/La Niña (Fig 1). Our meta-analysis included a total of 453 data points globally (Fig 3), which we subset into coral bleaching (n = 251) and coral cover (n = 202). The majority of data points were taken from graphs (n = 158); however, data points were also obtained from tables or directly from the text (n = 72).

The included studies employed a variety of survey types using both quadrat and transect methods, but in general these can be summarized into two categories: visual estimates and photo surveys. Included studies ranged in depth from 0.5m to 40m, although depth was often reported as a range nearly as large, hindering our ability to conduct any depth-specific analyses. We also were unable to examine taxonomic-specific effects of El Niño/La Niña-related bleaching analysis because even in the most commonly reported families (e.g. *Poritidae*, *Pocillloporidae*, *Acroporidae*) there were few (<20, generally <5–10) data points for each family. Additionally, most data points for each family were extracted from a maximum of 1–3 studies, which were often spatially clustered (e.g. *Pocilloporidae* on the Pacific coast of Central/South America). Similarly, we did not conduct analyses by biogeographic region or local sub-regions, as this is a broad-scale coral bleaching study focused on El Niño/La Niña-related warming events, and because we analyze underlying environmental factors (e.g. mean long-term temperature and variance) rather than coarse large-scale biogeographic regions. While we concede that factors such as depth and taxonomy are or may be important drivers of coral bleaching and cover changes during warming events, these specific analyses were not possible using currently available peer-reviewed research.

This meta-analysis focuses on peer-reviewed research on coral bleaching events which occur during El Niño and La Niña events. Although a great many other regional bleaching events have occurred outside of El Niño/La Niña events, the goal of this manuscript is to specifically investigate the influence of thermal stress on coral communities during El Niño and La Niña events. This may also, to some extent, limit the observed underlying environmental variability (e.g. by default our method excludes studies investigating bleaching due to cold water events, or point-source pollution). Additionally, if the "Web of Science" search is conducted similarly as above, but excluding the terms (El Niño OR El Nino OR ENSO), a total of 8,686 potential papers are returned for inclusion compared to the 773 reviewed in the current study. While this would be an admirable meta-analysis, it is outside of the scope of the current study.

### El Niño/La Niña effects on coral bleaching and coral cover

**Fig 4:**
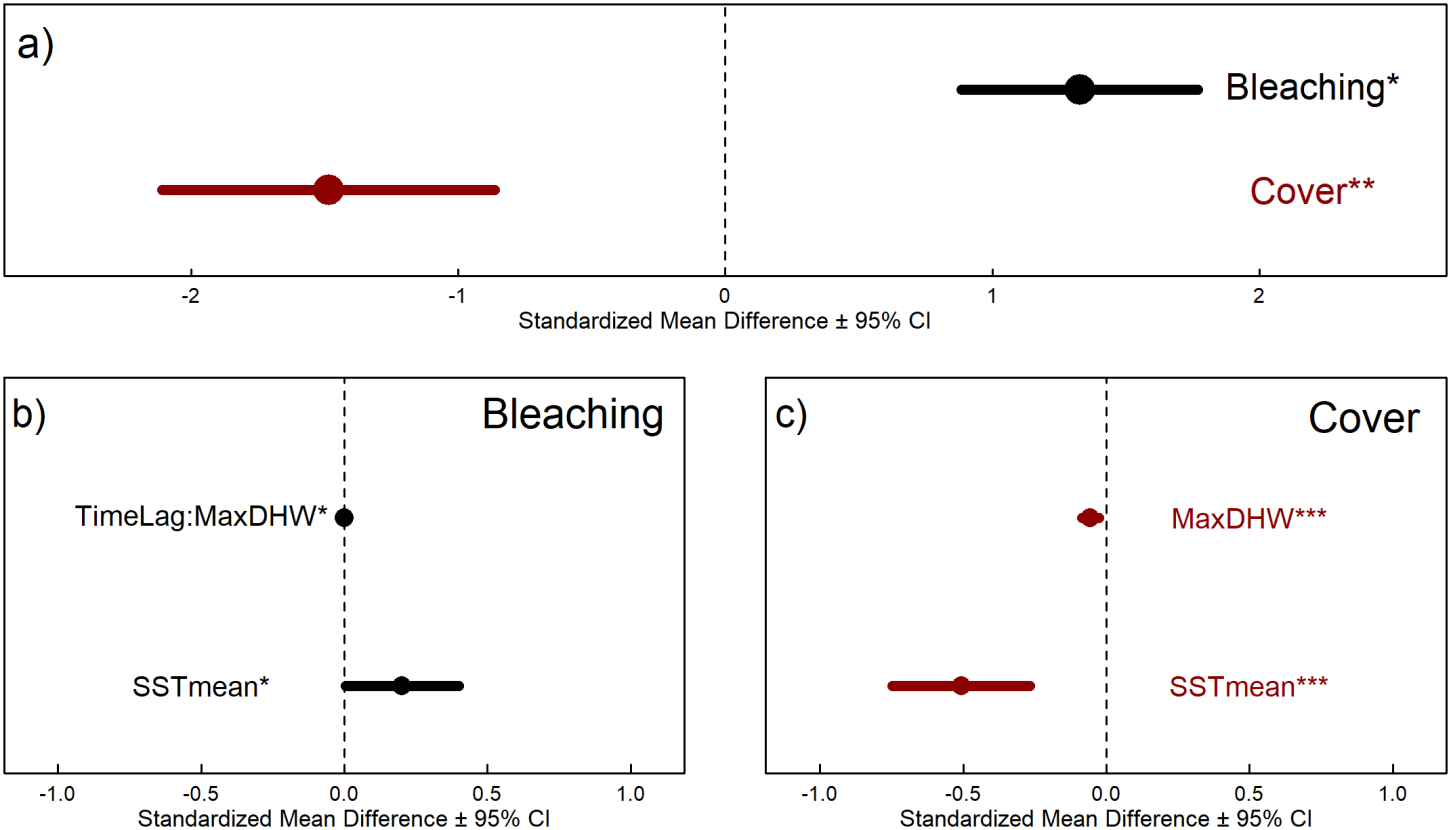
Effect size and moderators of top coral bleaching and coral cover models (p-value < 0.001 noted with ***, p-value <0.05 noted with *). a) Overall effect size (standardized mean difference ± 95% confidence intervals) for coral bleaching (black; including measured and simulated before-bleaching values) and coral cover loss (red; up to one year after maximum heat stress). El Niño/La Niña warming significantly increases coral bleaching and significant decreases coral cover. Significant moderators in b) the coral bleaching model and c) the coral cover model. MaxDHW is maximum DHW experienced by reef during the present El Niño event, SSTmean is the long-term mean temperature, and TimeLag is the time since maximum DHW occurred. A colon represents an interaction between two moderators.

As expected, El Niño- and La Niña-related warming causes an increase in coral bleaching (Fig 4a), with maximum coral bleaching reaching 100% in some locations. The best model for the partially-simulated before bleaching data set both using glmulti and reverse stepwise ANOVAs included long-term mean temperature, SSTmean (pseudo-R^2^ = 0.058, Table 2). Decreasing SSTmean lowered the effect of El Niño/La Niña heat stress on coral bleaching, with equatorial latitudes experiencing the most coral bleaching, and coral bleaching decreasing further away from the equator although this effect was small. We suggest that the most likely driver of this phenomenon is adaptation of corals to intra-annual temperature variability on higher latitude reefs [49]. Despite the fact that SSTmean accounts for a relatively small amount of variability (~6%, pseudo-R^2^ = 0.058), it is still notable that this moderator appears to influence coral bleaching, given the variety of other factors that also contribute to patterns in coral bleaching. When variability of effect sizes between different sites/time points within a paper were taken into account, TimeLag was removed as a significant moderator. This is most likely due to the fact that we expect the effect size (i.e. how much bleaching occurred) to vary within a study in direct relation to time since maximum heating occurred. Consequently, taking this into account in our model construction essentially masks the true effect of TimeLag on coral bleaching by accounting for this in within-study variation.

**Table 2:** Top model results for coral bleaching (including measured and simulated before-bleaching values) and coral cover loss (up to one year after maximum heat stress).

Model	Moderators (Top Model)	QM	df	QE	df
Bleaching	SSTmean, TimeLag:MaxDHW	7.5***	2	1652***	140
Cover	MaxDHW, SSTmean	21***	2	796***	153

QM is the test of moderators (i.e. whether the moderators explain a significant amount of variance in the model), and QE is the test for residual heterogeneity (unexplained variance) after accounting for all included variables (p-value < 0.001 noted with ***). MaxDHW is maximum DHW experienced by reef during the present El Niño event, SSTmean is the long-term mean temperature, and TimeLag is the time since maximum DHW occurred. A colon represents an interaction between two moderators. See S3(b) and S3(c) Fig for the top ten model results.

Overall, percent coral cover significantly decreased after an El Niño/La Niña event (Fig 4a). The maximum reduction of local reef cover among studies was 100%, which was due to taxa-specific losses estimated within studies (absolute loss across the entire coral community = 80.5%). The model moderators with the best consensus for explaining coral cover loss were MaxDHW and SSTmean (pseudo-R^2^ = 0.047, Fig 4c, Table 2). As expected, coral cover loss increased as cumulative heat stress (MaxDHW) increased (Fig 4b). Long-term mean temperature is also a significant moderator, with increasing SSTmean related to decreases in coral cover. Only the top two consensus moderators were included in the main presentation of results (Table 2, Fig 4). Although three of four coral cover models agreed on these two moderators, one model that included the full two years of data (after the El Niño/La Niña peak, constructed with glmulti) also included a small, but statistically significant interaction between MaxDHW and TimeLag (S3a Fig). The model including full two years of data (constructed with glmulti) did not include MaxDHW, although the other models (full two years of data constructed with glmulti, one year of data only and full two years of data constructed with reverse stepwise ANOVAs) did include MaxDHW as a significant moderator. Since SSTmean is a significant moderator in both bleaching and cover models, if we accept long-term mean temperature as a proxy for latitude, then low-latitude coral communities experience both higher bleaching and coral cover loss during El Niño/La Niña events (Fig 4) than corals at higher latitudes. However, the thermal gradient in coral cover loss we demonstrate in this meta-analysis (and the proposed corresponding decrease in coral loss with increasing latitude) is in contrast to a previously observed latitudinal gradient coral bleaching (increasing bleaching with increasing latitude) [71]. This lends support to the hypothesis of increased recovery at higher latitudes, where corals bleach more frequently, but are either acclimated or adapted to recover from bleaching stress.

### Residual heterogeneity

Within all models, there was a significant degree of heterogeneity remaining after accounting for all significant moderators (Table 2). This suggests additional factors not included within these models have a significant influence upon coral bleaching and cover loss during ENSO. This may also be due to non-linearities in moderator-response interactions, which we were unable to test with the available data. With more data, or with a more constrained question (e.g. local- or regional-scale analyses), exploring non-linear models may be instructive for understanding residual heterogeneity not resolved with linear modelling. Factors not included within our models that could account for this heterogeneity include coral taxa, local adaptation, and coral depth, as well as a suite of abiotic factors including wind, upwelling and currents, and localized (i.e. meter to kilometer scale) thermal anomalies. The intensity and distribution of coral bleaching can be affected by the taxonomic composition of individual reefs, based on species-specific bleaching susceptibilities [72]. Reef location, both depth and distance to shore, are also important factors determining the vulnerability of corals to thermal bleaching and subsequent mortality [73]. Local acclimation and adaptation were not considered in our study, but almost certainly influence coral bleaching patterns as well [74]. For example, local oceanographic conditions affect susceptibility to bleaching, as high water flow [75] and upwelling [76] potentially limiting acclimation and decreasing coral tolerance to warming. Many additional factors contribute to heterogeneity in coral bleaching patterns, ranging from cloud cover [77] and water quality [78, 79], to basic coral biology and micro-complexity [80]. These factors are fundamentally important to understanding patterns in coral bleaching, survival, and resilience, and continued investigation and synthesis are encouraged as more data become available.

### The novelty of the 2015–2016 El Niño event

**Fig 5:**
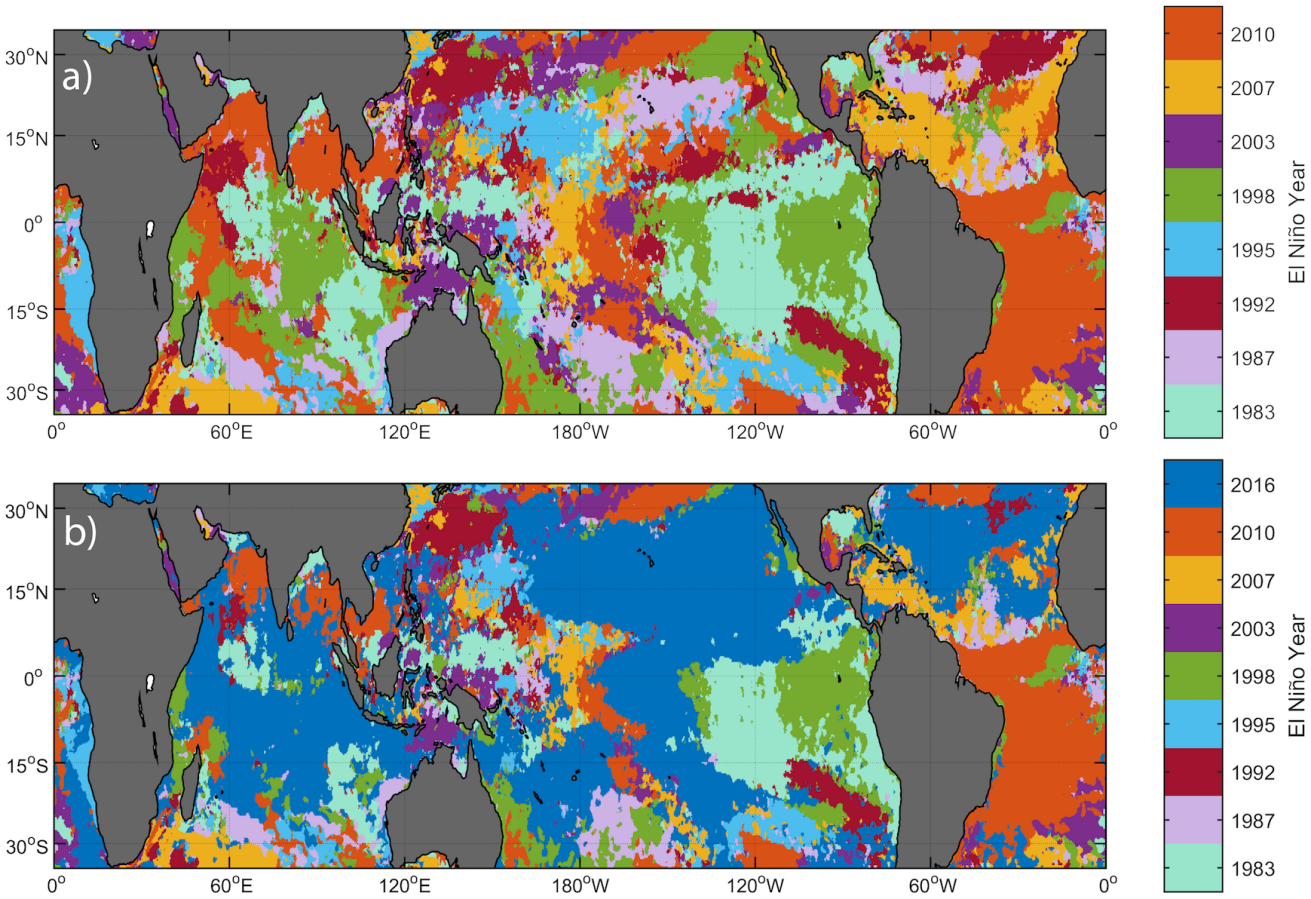
El Niño events with the greatest heat stress. Both figures show which El Niño event caused the greatest maximum DHW for each area. Note that this figure does not demonstrate bleaching response, only maximum cumulative heat stress per El Niño event. The events are color-coded by year. The 1997/1998 El Niño event (green) was the most severe event in the Eastern Pacific around the South American coast. a) All El Niño events from 1982–2010, showing how much heterogeneity there is in the geographic distribution of the most extreme heat stress. b) All El Niño events since 1982, including the 2015–2016 El Niño event, demonstrating the coral heat stress homogenization that occurred during this most recent El Niño/La Niña warming event.

Prior to 2015, the most extreme El Niño events observed to impact coral reefs globally occurred in 1982–83 and 1997–98. The 2015–2016 El Niño surpassed these events both in terms of ocean warming intensity and extent [81], causing unprecedented ecological consequences worldwide. While areas affected by typical Eastern Pacific El Niño events (i.e. the coast of Central America) were still not affected worse than the massive El Niño in 1997–1998, the 2015–2016 El Niño now dominates tropical waters as the highest cumulative stress on corals globally on record (Fig 5). In fact, for several reefs in the Central Pacific region, the 2015–2016 El Niño exceeded the threshold "Not experienced by reefs as yet" (24 Degree Heating Weeks) described by Hoegh-Guldberg merely 6 years ago [82]. As the return time between bleaching events decreases, we expect that the balance of long-term influence of coral resistance versus recovery from bleaching events will shift, making coral resistance incrementally more important than recovery [83].

### Recommendations

Based on the outcomes of this systematic review of the published ENSO-warming related coral literature, we make several recommendations for future coral reef bleaching studies. First, researchers should include at least the following El Niño/La Niña warming parameters:

- Magnitude of warming: current local Degree Heating Week at the time of field sampling;
- Timing and trajectory of warming (e.g. include figure with temperature or DHW trajectory leading up to sampling time point);
- A history of bleaching events for the study location(s). We suggest including a supplementary figure of heat stress over time since 1982 (e.g. by extracting data from our new DHW data product). This would allow for examination of historic heat stress, as well as the potential for local acclimation and adaptation due to previous heat stress conditions.

Additionally, it is imperative that researchers present all study parameters including:

- Exact sampling dates for each location sampled;
- Exact GPS coordinates for each location sampled, including multiple GPS coordinates in cases where there is more than one study site;
- Sample size, at the smallest measured scale (e.g. samples per site);
- Sampling error (e.g. standard deviation or standard error);
- Any available before-impact data: coral cover, coral bleaching, including corresponding sampling errors, dates, and methods;
- Exact coral survey depth(s). Large depth ranges (e.g. 5- 20m depth), and even moderate ranges at critical depths (e.g. 2-10m depth) obfuscate patterns in coral bleaching by greatly increasing unexplained variability in light exposure. If a study examines coral bleaching at different depths, this should be reported, with data and results specified by depth.

As well as study-specific taxonomic information including:

- Taxonomic composition of the surveyed corals, as well as species-specific coral responses to heat stress;
- Overall coral community response, in cases where the primary purpose of the study is to investigate single (or a few) coral species.

Finally, we encourage researchers to make their data and results fully open upon publication of their study. This includes, but is not limited to: photo/video survey methods which can produce archives of coral reef status. Reproducibility, and consequently future synthesis work, would be enhanced by making images available online (through sites like CoralNet [84]), and by making analyses transparent by providing data, code, and results (via sites like GitHub).

We found that only a subset of papers considered for this meta-analysis included both the temperature stress (i.e. either temperature anomaly at the time of sampling or cumulative DHW) and a specific time period of when the sampling took place. It will be much easier to identify patterns in bleaching and mortality if we can rectify the data we already have to a quantitative time frame of thermal stress. Additionally, we found that 20 out of 44 coral bleaching papers and 20 out of 61 coral cover papers did not include sample size, sampling error, or both. Finally, we note that long-term monitoring data sets are important, as they provide a baseline against which to compare changes to coral reef ecosystem structure and health, and we strongly support the development of such data sets. Building a mechanistic understanding of how local variability in baseline coral cover and bleaching changes during El Niño/La Niña warming will allow us to identify the processes that give rise to bright spots [85] that foster coral reef resilience and recovery over the long term.

### Conclusion

Understanding how El Niño/La Niña events impact coral reefs is crucial for developing strategies for coral reef conservation, which is important not only for biodiversity conservation, but also because tens of millions of people in over 100 countries rely on coral reefs for subsistence and to maintain their livelihoods [86]. The additional benefits that coral reefs provide are extensive, including protection against wave action, provision of fish habitat [87], recreation and tourism, and aesthetic and cultural benefits [88–90]. The resilience of these benefits is incrementally being eroded, as local stressors decrease baseline resilience [91,92] and climate change disables coral bleaching protection by shifting ocean warming trajectories on reefs from “protective” (trajectories that include a moderate amount of warming followed by a period of recovery before more intense heating instigates a bleaching event, essentially priming the corals to better respond to the heat), to “lethal” (trajectories that either spike rapidly and/or remain above bleaching thresholds) [93]. Our meta-analysis confirms that El Niño and La Niña-associated heat stress, as measured by maximum DHW, is a likely contributor to patterns of coral cover loss across the world's oceans. We also found that this trend is mediated by a temperature gradient in ENSO-associated coral cover loss, suggesting that higher latitude reefs may experience a smaller amount of El Niño and La Niña-associated decline compared to equatorial reefs. We show that there is a dearth of published studies reporting taxa-specific responses and changes in broad-scale ecosystem metrics to El Niño and La Niña, and we recommend that future studies should incorporate a broader range of resilience metrics in order to cope with measurement uncertainty and ecological surprise [94]. Future syntheses of recent and emergent bleaching events will allow us to discover where reefs are doing better than expected, and to more accurately focus global research, management, and conservation efforts.

## Supporting information

S1 Table Overview of all papers reviewed for the meta analysis. ACC is accession number, which is unique to each manuscript/study returned from the Web of Science search. Some manuscripts were duplicates of previous ACCs, which is noted in the 'Duplicate of' column. Manuscript metadata included authors, year of publication, manuscript title, and journal name, number, and pages. The 'Source' column notes where each manuscript was initially found (e.g. Web of Science). Relevancy criteria were assessed in the columns 'Initial Possibly Relevant', 'Possibly Relevant', 'Provides.info.on.coral.responses.bleaching.cover.OR.mortality', 'Compares coral responses during or post El Nino_general', and 'Compares coral responses w El Nino', and 'Include' notes which papers were included in the final data set. Other reasons for not including papers in the final data set were that they were inaccessible ('Cant.Access'), they had no sample size or standard deviation/error measurement ('No N or SD?','NoNorSD'), the study did not occur during/after El Niño/La Niña stress ('Not.El.Nino'), the study was not published in the primary literature ('NotPrimaryLit'), the manuscript was a review or meta analysis ('Review.MetaAnalysis'), the data was repeated in previous manuscripts ('Data.elsewhere'), coral cover was listed without a before-impact measurement ('Cover.no.before'), there was no extractable data ('No.extractable.data'), measurements were qualitative ('Qualitative'), units were measured in density (e.g. colonies/unit area; 'Units are Density'), recovery measurement occured more than two years after the El Niño/La Niña impact ('Recovery (2+ years)') or 'Other reason for not including'. Study type is specified in the columns 'Cover', 'Cover.Extracted', 'Bleaching', 'Bleaching.Wbefore', and 'Bleaching.AfterOnly'.
(CSV)

S2 Table All coral cover data included in the meta-analysis. ACC is accession number, which is unique to each manuscript/study returned from the Web of Science search. Manuscript metadata included author, year, page number, and which parameter was measured in the study. Coral taxonomic data included whether hard or soft coral were measured ('Hard.Soft.Coral'), the order of coral studied, the coral family, genus, and species. Survey method is described in 'Method' and 'Method.simple', and survey date information is located in 'Month.Before', 'Month.During.After', 'Year.Before', 'Year.During.After', 'Day.Sampled', 'Month.Sampled', 'Year.Sampled', 'Date.Sampled', and 'Date.Sampled.Type'. Which El Niño/La Niña event event was surveyed is recorded in 'El.Nino', 'El.Nino.simple', 'La.Nina', and 'La.Nina.simple'. Measurement time periods are denoted in 'Measured.Before', 'Measured.During', 'Measured.After.0.6months', 'Measured.Duringto6mo', and 'Measured.After.6months.2years'. Depth and location data are located in 'Depth', and 'Lat.dec', 'Lat.abs', 'Long.dec', 'Ocean', 'Region', 'Ocean.Region.simple', and 'Location'. Data extraction details included where in the manuscript the data was extracted from ('Source'), whether data was extracted using Graph Click or from the text/table (Y and N respectively, 'Graphclick'), what the originally measured units are ('Units.original'). Measured values, error, and sample size are reported in 'Mean_Cover_Before', 'SE_Cover_Before', 'SD_Cover_Before', 'Mean_Cover_Resp', 'SE_Cover_Resp', 'SD_Cover_Resp', 'Mean_Bleaching_Before', 'SE_Bleaching_Before', 'SD_Bleaching_Before', 'Mean_Bleaching_Resp', 'SE_Bleaching_Resp', 'SD_Bleaching_Resp', 'N_Before', and 'N_During.After'. Derived satellite data are defined in Table 1, and include the maximum degree heating week experienced at the time of sampling ('MaxDHW'), time between maximum degree heating week and sampling period ('TimeLag'), long-term mean temperature ('SSTmean'), and long-term temperature variability ('SSTvar').
(CSV)

S3 Table Coral bleaching after-only data included in the meta-analysis. ACC is accession number, which is unique to each manuscript/study returned from the Web of Science search. Manuscript metadata included author, year, and which parameter was measured in the study. Coral taxonomic data included whether hard or soft coral were measured ('Hard.Soft.Coral'), the order of coral studied, the coral family, genus, and species. Survey method is described in 'Method', and survey date information is located in ''Month.Before', 'Month', 'Month.During.After', 'Year.During.After', 'Day.Sampled', 'Month.Sampled', 'Year.Sampled', 'Date.Sampled', and 'Date.Sampled.Type'. Which El Niño/La Niña event was surveyed is recorded in 'El.Nino', 'El.Nino.simple', 'La.Nina', and 'La.Nina.simple'. Measurement time periods are denoted in 'Measured.During', 'Measured.After.0.6months', 'Measured.Duringto6mo', and 'Measured.After.6months.2years'. Depth and location data are located in 'Depth', and 'Lat.dec', 'Lat.abs', 'Long.dec', 'Ocean', 'Region', 'Ocean.Region.simple', and 'Location'. Data extraction details included where in the manuscript the data was extracted from ('Source'), whether data was extracted using Graph Click or from the text/table (Y and N respectively, 'Graphclick'), what the originally measured units are ('Units.original'). Measured values, error, and sample size are reported in 'Bleaching.Level', 'Mean_Bleaching_Before' (simulated), 'SE_Bleaching_Before' (simulated), 'SD_Bleaching_Before' (simulated), 'Mean_Bleaching_Resp', 'SE_Bleaching_Resp', 'SD_Bleaching_Resp', and 'N_During.After'. Derived satellite data are defined in Table 1, and include the maximum degree heating week experienced at the time of sampling ('MaxDHW'), time between maximum degree heating week and sampling period ('TimeLag'), long-term mean temperature ('SSTmean'), and long-term temperature variability ('SSTvar').
(CSV)

S1 Fig PRISMA (Preferred Reporting Items for Systematic Reviews and Meta-Analyses) 2009 Checklist. Official PRISMA checklist, with criteria and corresponding manuscript pages.
(PDF)

S2 Fig Animation of progression of maximum El Niño impact by event, from 1982 to 2016. Animated progression through El Niño events from 1982 to 2016, showing which El Niño event caused the maximum thermal impact (degree heating weeks) for all grid cells.
(MP4)

S3 Fig All model results. a. Moderators included in top models. Output from the R package 'glmulti', as well as reverse stepwise ANOVAs are included, and two data sets were included for each method (all coral cover data, and only data collected within 1 year of the peak El Niño/La Niña event. Moderators are denoted as either positive (+) or negative (-). A dark grey background indicates that the moderator was not included in the final model and white background indicates that the moderator was included in the final model and was significant at p < 0.05. b. Top ten best models for coral cover, showing included model terms (moderators), AICc values and weights. c. Top ten best models for coral bleaching, showing included model terms (moderators), AICc values and weights.
(DOC)
