# Supplementary material for: Global patterns and impacts of El Niño events on coral reefs: A meta-analysis
Source: PLoS One. 2018 Feb 5;13(2):e0190957. doi: 10.1371/journal.pone.0190957 (PMC5798774; doi:10.1371/journal.pone.0190957)
Supplement: S3 Fig — a. Moderators included in top models. Output from the R package 'glmulti', as well as reverse stepwise ANOVAs are included, and two data sets were included for each method (all coral cover data, and only data collected within 1 year of the peak El Niño/La Niña event. Moderators are denoted as either positive (+) or negative (-). A dark grey background indicates that the moderator was not included in the final model and white background indicates that the moderator was included in the final model and was significant at p < 0.05. b. Top ten best models for coral cover, showing included model terms (moderators), AICc values and weights. c. Top ten best models for coral bleaching, showing included model terms (moderators), AICc values and weights. (DOC) [file pone.0190957.s006.doc]

| Model Method | Included Data Type | Mean Temp | Max DHW | Max DHW:  TimeLag |
| --- | --- | --- | --- | --- |
| glmulti | Cover, All Data | - | x | - |
|  | Cover, 1 Year Only | - | x | x |
| Reverse Stepwise ANOVAs | Cover, All Data | - | - | x |
|  | Cover, 1 Year Only | - | - | x |
| glmulti | Bleaching, collected + simulated “before” data | + | x | - |
|  | Bleaching, only simulated “before” data | x | x | - |
| Reverse Stepwise ANOVAs | Bleaching, collected + simulated “before” data | + | x | x |
|  | Bleaching, only simulated “before” data |  |  |  |

1. Moderators included in top models
2. Coral Cover (up to 1 year after maximum stress).

| **Included Model Terms** | **AICc** | **Weight** |
| --- | --- | --- |
| Max DHW + Mean Temp (Top Model) | 552.70 | 0.0358 |
| Mean Temp + Mean Temp:Max DHW | 552.87 | 0.0330 |
| Max DHW + Temp Var | 553.48 | 0.0242 |
| Temp Var + Mean Temp:Max DHW | 553.56 | 0.0232 |
| Max DHW + Temp Var:Mean Temp | 553.69 | 0.0218 |
| Mean Temp:Max DHW + Temp Var:Mean Temp | 553.70 | 0.0217 |
| Max DHW + Mean Temp + Time Lag:Max DHW | 554.02 | 0.0185 |
| Max DHW + Mean Temp + Temp Var:Mean Temp | 554.04 | 0.0184 |
| Max DHW + Mean Temp + Mean Temp:Time Lag | 554.05 | 0.0183 |
| Max DHW + Time Lag + Mean Temp | 554.07 | 0.0181 |

1. Bleaching (including measured and simulated before-bleaching values)

| **Included Model Terms** | **AICc** | **Weight** |
| --- | --- | --- |
| Mean Temp + Time Lag:Max DHW | 433.64 | 0.0377 |
| Time Lag + Mean Temp | 434.33 | 0.0267 |
| Mean Temp + Mean Temp:Time Lag | 434.39 | 0.0260 |
| Mean Temp + Temp Var + Time Lag:Max DHW | 434.59 | 0.0235 |
| Mean Temp + Time Lag:Max DHW + Temp Var:Mean Temp | 434.63 | 0.0230 |
| Mean Temp + Temp Var + Temp Var:Time Lag | 434.83 | 0.0208 |
| Mean Temp + Temp Var: Time Lag + Temp Var:Mean Temp | 434.84 | 0.0207 |
| Mean Temp + Temp Var:Time Lag | 434.91 | 0.0200 |
| Time Lag + Mean Temp + Mean Temp:Time Lag | 435.42 | 0.0155 |
| Time Lag + Mean Temp + Temp Var | 435.43 | 0.0154 |
